# Physical activity and its effects in myasthenia gravis: a patient-reported study on habits and impact

**DOI:** 10.1186/s12883-025-04590-x

**Published:** 2025-12-17

**Authors:** Stefanie Glaubitz, Stefanie Meyer, Johanna Loris, Rachel Zeng, Karsten Kummer, Tania Garfias-Veitl, Ariana Seraji, Lina Hassoun, Denise Rohleder, Ina Hunger, Jana Zschüntzsch

**Affiliations:** 1 Department of Neurology, University Medical Center Göttingen, Robert-Koch-Straße 40, Göttingen, 37075 Germany; 2 Heart & Brain Center Göttingen, University Medical Center Göttingen, Göttingen, Germany; 3 Department of Neurology, Faculty of Medicine and University Hospital Carl Gustav Carus, TUD Dresden University of Technology, Dresden, Germany; 4 Department of Neurology and Pain Treatment, Immanuel Clinic Rüdersdorf, University Hospital of the Brandenburg Medical School Theodor Fontane, Rüdersdorf bei Berlin, Germany; 5 Department of Medical Statistics, University Medical Center Göttingen, Göttingen, Germany; 6 Department of Neurology, KRH Klinikum Nordstadt, Hannover, Germany; 7 Institute of Sport, University Bremen, Bremen, Germany

**Keywords:** Myasthenia gravis, Physical activity, Exercise, QMG, Quality of life, MG-ADL

## Abstract

**Background:**

Physical activity and exercise have been classified as safe and effective in mild to moderate cases of myasthenia gravis (MG) across various studies. Subsequently, adequate physical activity is generally recommended. Nevertheless, individuals with MG remain less physically active than the general population, without a precise definition of the low-activity group so far.

**Methods:**

In this prospective single-center study, individuals with MG completed a questionnaire assessing general mobility, weekly physical activity levels, and beliefs toward specific statements about physical exercise. These data were contextualized with clinical parameters and MG-specific scores.

**Results:**

Among 84 individuals (50% female), 73.8% reported general positive effects, and 77.4% noted improvements in mood and well-being due to physical activity. No significant differences in physical activity levels were found depending on sex, BMI or age. Weekly physical activity averaged 94.6 min (SD: 85.6), falling below current recommendations. Physical activity was inversely correlated with lower QMG (*p* = 0.019) and MG-ADL scores (*p* = 0.004). Despite the reported positive impact of physical activity on quality of life, no relevant connection was detected between physical activity and MG-QoL15 scores.

Barriers preventing individuals affected by MG from engaging in physical activity included muscle pain (35.4%) and motivational challenges (22%). Individuals with motivational problems were younger (mean age 55.5 vs. 66.6 years, *p* = 0.011) and more frequently reported depressive symptoms; no other significant differences were observed in gender or disease severity in this subgroup.

**Conclusion:**

Individuals with MG perceive physical activity as beneficial to their physical well-being, mood, and overall quality of life. Those with less severe disease tend to be more active. However, barriers such as motivational issues and post-exercise pain must be addressed. Clinicians should aim to identify individuals with low activity levels, encourage engagement in physical activity, highlight its benefits, and alleviate patient concerns.

**Trial registration:**

Study approval by the Ethics Committee of the University Medical Center Göttingen was granted (number 33/12/21). The study was retrospectively registered at the German Clinical Trial Registry (DRKS) under the study ID DRKS00033171 (Date of trial registration December 1st, 2023).

**Supplementary Information:**

The online version contains supplementary material available at 10.1186/s12883-025-04590-x.

## Background

The health benefits of physical activity are widely acknowledged, including a reduced risk of mortality [1]. Current recommendations by the World Health Organization (WHO) for healthy adults include performing at least 150–300 min of moderate-intensity aerobic physical activity a week, or 75–150 min of vigorous-intensity activity, in addition to muscle-strengthening activities on two or more days of the week [2]. The same recommendations apply to individuals aged 65 years and older as well as to those affected by chronic conditions; however, they are instructed to focus increasingly on functional balance and strength training to enhance functional capacity and reduce fall risk. According to the WHO, physical activity encompasses any movement of the body generated by skeletal muscles that leads to energy expenditure. It includes all kinds of movement, such as activities performed during leisure time, while commuting or traveling from one place to another, as well as tasks carried out at work or in the home [3]. In contrast, exercise is a specific type of physical activity that is intentional, organized, and repetitive, with the primary or secondary goal of enhancing or maintaining physical fitness [4].

In autoimmune diseases, regular physical activity has been associated with improvements in cardiovascular comorbidities as well as enhanced health-related quality of life [5].

Myasthenia gravis (MG) is a rare, chronic autoimmune disorder characterized by impaired neuromuscular transmission, resulting in fluctuating and exertion-induced muscle weakness. Commonly affected muscle groups include the ocular, facial, axial, and limb musculature [6]. Current standard treatment strategies for MG primarily involve pharmacological interventions and thymectomy [7, 8]. Over the past several years, the therapeutic landscape has further evolved with the introduction and approval of novel, highly effective immunomodulatory agents [9–12].

Beyond pharmacological treatment, the benefits of exercise on disease-related parameters in MG are increasingly recognized. Both observational and interventional studies have explored this topic, using aerobic, resistance, and balance training interventions [13–15]. However, the intensity, frequency, and duration of these interventions varied widely depending on the individual study design, reducing comparability and inhibiting derivation of commonly accepted therapeutic recommendations. Common outcome measures included the six-minute walk test, adverse events, muscle strength, MG-specific quality of life (MG-QoL-15), forced vital capacity, the Quantitative Myasthenia Gravis Score (QMG), and the MG Activities of Daily Living Scale (MG-ADL) [13].

Current evidence indicates that exercise is safe for individuals with mild to moderate MG [13–15]. Therefore, standard exercise guidelines, recommending at least 150 min of exercise per week, are considered appropriate for individuals affected by MG [14]. To date, studies investigating physical activity patterns in individuals with MG have consistently demonstrated that individuals with MG are less physically active compared to healthy controls [16, 17]. Prior research, employing both self-reported questionnaires [18, 19] and objective measurements using accelerometers [16, 17, 19], has reported that only a small proportion of individuals with MG meet general recommendations for daily step count and physical activity levels [16–19]. Notably, most studies have failed to identify a consistent association between disease severity and the extent of physical activity [16, 17], suggesting that other contributing factors to reduced activity levels need to be explored.

To our knowledge, the field has not yet saturated the use of MET-based analysis. Integrating MET minutes with symptom scales, demographic moderators, and qualitative barrier assessment constitutes a novel, needed direction to refine exercise recommendations in MG. The present study aims to evaluate the level of exercise, the types of exercise performed, and individuals’ perceived impact of physical activity on general well-being, quality of life, and MG-related symptoms. In addition, the study seeks to explore how disease severity, demographic variables (e.g., age and sex), and potential barriers, such as post-exertional myalgia, muscular weakness, and motivational challenges, affect exercise engagement in this population.

## Methods

### Study design

This prospective, single-centre study was conducted at the University Medical Centre Göttingen (UMG) from February 2022 to February 2024. Approval was granted by the Ethics Committee of the University Medical Centre Göttingen on 5th January 2022 (ethics number: 33/12/21). The study was retrospectively registered at the German Clinical Trial Registry (DRKS) under the study ID DRKS00033171 (Date of trial registration: December 1st, 2023). Written consent was obtained from all participants.

The participating individuals were treated on the neurological wards, in the day-care clinic, in the infusion outpatient clinic and in the neuromuscular outpatient clinic at the Department of Neurology (UMG).

The inclusion criteria encompassed individuals over the age of 18 years, with the capacity to provide consent, and a diagnosis of MG in accordance with national guidelines [20]. Individuals were asked to complete a study-specific questionnaire on physical activity. In addition, a comprehensive set of demographic and clinical data was systematically collected, including sex, age, smoking status, body weight, and height. Disease-specific information was also gathered, encompassing key clinical parameters related to MG. These included assessments using the German version of MG-ADL [21], the QMG [22], and the German version of MG-QoL15 [23].

Further data extracted from health records comprised antibody status, disease duration, history of thymectomy and thymoma, as well as details on symptomatic and immunosuppressive treatments. The secondary diagnoses were also collected using the patient files and were not queried separately.

### Study-specific questionnaire

The study-specific questionnaire included three sections and was developed for this study (Supplement 1, Study-specific questionnaire). The questionnaire was answered in German and translated to English for publication. The first section focused on mobility, asking about maximum and average daily walking distance, mobility at home and in public, stair climbing ability, and use of assistive devices. The second section covered physical activity habits, including frequency, duration, and types of exercise, with an open-text field. Participants were encouraged to report even light exercise. The third section presented 12 statements rated on a five-point Likert scale: “strongly agree”, “mostly agree”, “undecided”, “mostly disagree”, and “strongly disagree”.

### Scales and definitions

To enable mapping and comparison of exercise, the metabolic equivalent of task (MET) for each exercise and physical activity was recorded based on the 2024 Compendium of Physical Activities [24]. The MET value facilitates the comparison of various activities by quantifying their energy expenditure. One MET is defined as the consumption of one kilocalorie per kilogram of body weight per hour, corresponding to the resting metabolic rate. Moderate physical activity typically ranges from 3 to 6 METs, while intensive physical activity exceeds 6 METs [24].

Because individuals engage in various exercise activities with differing durations and frequencies to allow inter-individual comparison, an average MET value per individual was calculated, combining duration and frequency. For example, if an individual reports activities with MET values of 2 and 4, the average MET is 3. If performed twice weekly for one hour each, the weekly MET is 6 (2 sessions × 1 h × 3 MET).

Quartiles based on MET values were used to stratify individuals into four groups. The top and bottom 25% were compared on specific outcomes. Individuals who reported no exercise were assigned to quartile with the lowest activity.

For agreement with statements, “strongly agree” and “mostly agree” were grouped as agreement, while “mostly disagree” and “strongly disagree” indicated disagreement. Changes in disease activity were inferred from medication adjustments, such as increased doses of acetylcholinesterase inhibitors, glucocorticoids, or immunoglobulins since the last evaluation.

### Statistical analyses

If not indicated differently, results are shown as mean ± standard deviation. The unpaired t-test was used to compare parameters that were normally distributed, for example in the comparison of activity duration and intensity by gender. One-way ANOVA was used to compare several parametric values. The chi-squared test was used to examine the relationship between categorical variables. Fisher’s exact test was used to calculate p-values for categorical variables. Spearman rank correlation analysis was performed to analyze non-parametric data. All statistical analyses were conducted with a significance level of α = 5% (*p* ≤ 0.05). As this was an exploratory study, no correction for multiple testing was applied. All statistical analyses were conducted using *GraphPad Prism version 8 (La Jolla*,* CA*,* USA*, www.graphpad.com).

## Results

### Baseline characteristics

A total of 84 individuals diagnosed with MG were included in the study, with an equal gender distribution (50% female). The mean age was 62.2 years (± 14.8), and the majority (*n* = 76; 90.5%) were diagnosed with generalized MG. The average disease duration was 8.7 years (± 8.9). Regarding antibody status, 12 individuals (14.3%) were seronegative. The acetylcholine receptor antibody (AChR-ab) was detected in 82.1% of individuals, followed by anti-titin antibodies, which may be unspecific in an elderly patient population, in approximately one third (*n* = 26; 31%). A combination of different antibodies was present in 29.8% of cases.

All individuals received symptomatic treatment with acetylcholinesterase inhibitors. Approximately two thirds (*n* = 53, 63.1%) were on corticosteroid therapy. Immunosuppressive therapy other than glucocorticoids was used in 66.7% of individuals, primarily azathioprine, followed by mycophenolate mofetil. Nine individuals received complement inhibitors or FcRn antagonists. Intravenous immunoglobulin (IVIG) therapy was administered in 21.4% (*n* = 18). Regarding the myasthenia gravis-specific scores, the QMG was 8.5 ± 5.7 points, the MG-ADL was 8.4 ± 4 points, and the MG-Qol-15 was 18.1 ± 14.1 on average. An overview of the baseline characteristics data can be found in Table 1.

**Table 1:** Basic characteristics

Characteristics	Cohort (*n* = 84)
Baseline Characteristics	
Female, n (%)	42 (50)
Body mass-Index, (kg/m^2^), mean (SD)	28.3 (5.9)
Non-Smoker, n (%)	70 (83.3)
Disease specific characteristics	
Age at survey, year, mean (SD)	62.2 (14.8)
Age at diagnosis, year, mean (SD)	53.7 (18.5)
Disease duration, year, mean (SD)	8.7 (8.9)
EOMG, n (%)	26 (31)
Generalized MG, n (%)	76 (90.5)
Ocular MG, n (%)	8 (9.5)
History of thymectomy, n (%)	23 (27.3)
Confirmed thymoma, n (%)	10 (11.9)
MG crisis in past (at least one), n (%)	14 (16.7)
Antibody status, n (%)	
Anti-AChR-Ab	44 (52.4)
Anti-MuSK-Ab	2 (2.4)
Anti-Titin-Ab	1 (1.2)
AChR-Ab + Titin-Ab	23 (27.4)
AChR-Ab + MuSK-Ab + Titin-Ab	2 (2.4)
Seronegative	12 (14.3)
MG specific Scores, mean (SD)	
QMG	8.5 (5.7)
MG-ADL	4.8 (4)
MG-QoL15	18.1 (14.1)
Therapy, n (%)	
Pyridostigmine	84 (100)
Glucocorticosteroids	53 (63.1)
Azathioprine	29 (34.5)
Mycophenolate mofetile	14 (16.7)
Methotrexate	4 (4.8)
Efgartigimod	4 (4.8)
Rosazanolixizumab	1 (1.2)
Zilucoplan	2 (2.4)
Eculizumab	2 (2.4)
IVIG	18 (21.4)

Disease duration comprises the interval from diagnosis to study. EOMG is defined by symptom onset prior to the age of 50 years

*Abbreviation: Ab* antibody, *AChR* acetylcholine receptor, *EOMG* early onset myasthenia gravis, *IVIG* intravenous immunoglobulin, *MG* myasthenia gravis, *MG-ADL* Myasthenia Gravis Activities of Daily Living, *MG-QoL15* MG Quality of Life 15-Item Questionnaire, *min* minimum, *MuSK* muscle-specific tyrosine kinase, *n* number, *QMG* Quantitative Myasthenia Gravis, *SD* standard deviation

### Level of activity in the study cohort

Individuals reported a wide range of daily walking distances, averaging 3.0 (SD: 2.7) kilometers. Maximum walking distances showed a similar range. Most individuals did not require assistive devices in public areas and reported being able to ascend more than three flights of stairs; 50% used a handrail when climbing stairs.

Regarding exercise, 14 individuals reported to not perform exercise on any day of the week. However, about one third of study participants (*n* = 26, 31%) engaged in exercise on at least four days per week. The majority (*n* = 26, 37.1%) exercised for 30 to 60 min per session. Average weekly exercise duration was 94.6 min (range: 0–270; SD: 85.6), and 25% met the WHO recommendation of 150 min per week. Six individuals (7.1%) met the WHO recommendation for muscle-strengthening activities, which are advised to be performed on at least two days per week. Reported exercises included bodyweight-training and equipment training.

Walking was the most frequently reported activity (25.9%), followed by cycling (15.7%), seated gymnastics (12%), equipment training (6.5%), and gardening (6.5%). The mean modified weekly activity level was 7.40 MET* (range: 0–44.2). An overview is provided in Table 2.

**Table 2:** Mobility and exercise in individuals with MG (*n* = 84)

Mean walking distance, kilometers (SD)	
Mean daily walking distance (self-reported)	3.0 (2.7)
Mean maximum distance walked in one go (self-reported)	2.5 (3.0)
Walking or mobility aids in public areas, n (%)	
None	69 (82.1)
Hand stick	7 (8.3)
Walking frame	5 (6)
Wheelchair	3 (3.6)
Climbing stairs, n (%)	
No floor	2 (2.3)
One floor	16 (19)
1–2 floor	21 (25)
2–3 floors	18 (21.4)
> 3 floors	27 (32.1)
Aid for climbing stairs, n (%)	
None	36 (42.9)
Handrail	42 (50)
Handrail and support	5 (6)
Stair lift	1 (1.2)
Frequency of exercise per week, n (%)	
Never	14 (16.7)
One day	14 (16.7)
2–3 days	25 (29.8)
3–4 days	5 (6.0)
> 4 days	26 (31.0)
Duration of exercise session in individuals with activity (*n* = 70), n (%)	
< 10 min	5 (7.1)
< 20 min	12 (17.1)
20–30 min	13 (18.6)
30–60	25 (37.1)
> 60 min	14 (20.0)
Mean total weekly duration of exercise, min. (SD)	94.6 (85.6)
MET*-Score, n (%)	7.4 (8.7)

MET*-Score means a modified metabolic equivalent (calculated from the average MET value multiplied by frequency and duration of exercise per week)

*Abbreviations: min* minutes, *n* number, *SD* standard deviation

### Effects and concerns regarding physical activity and exercise

Regarding the effects on quality of life and general well-being, 73.8% of individuals reported that physical activity and exercise was beneficial overall, while 13.1% disagreed. Additionally, 77.4% noted improved mood and enhanced quality of life after engaging in physical activity and exercise, whereas 13.1% disagreed. However, only 42.9% reported a positive impact on physical symptoms, and 23.8% were undecided.

**Fig. 1:**
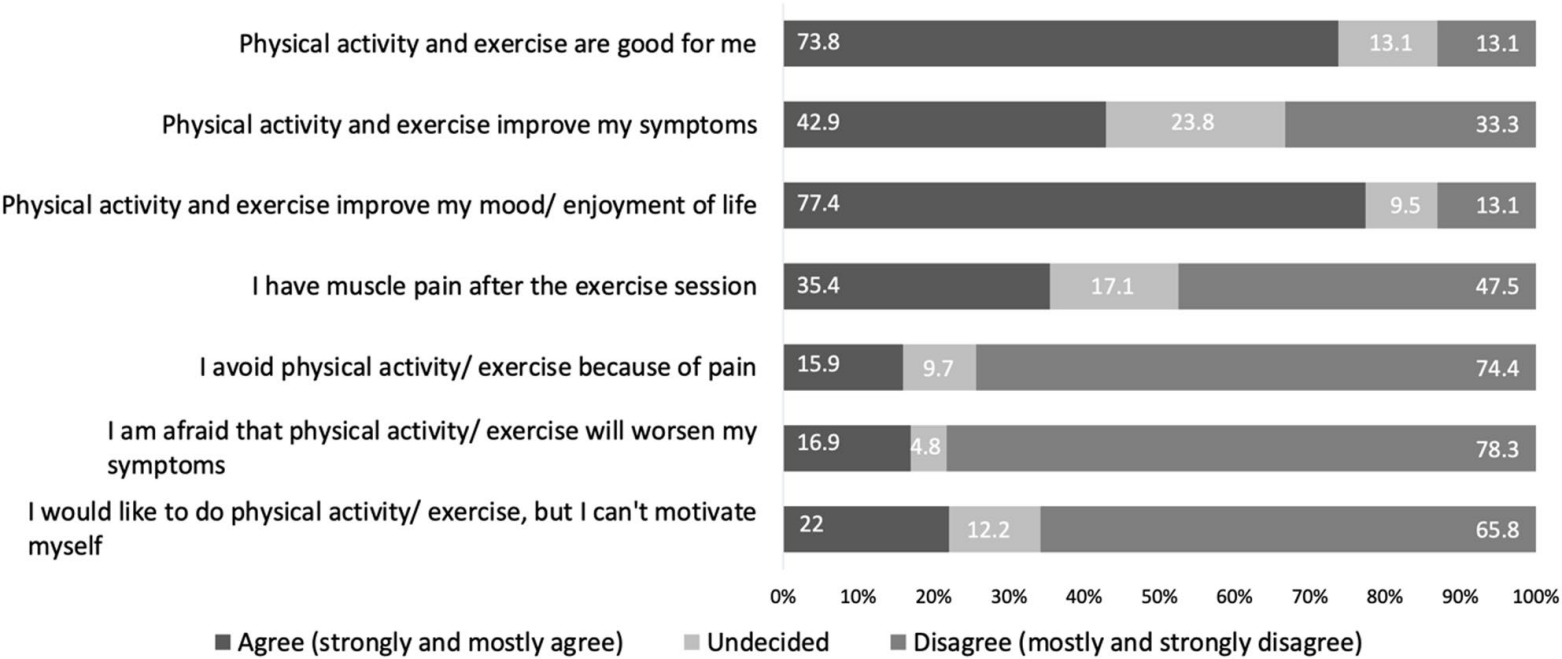
Responses to different selected statements regarding physical activity and exercise (*n* = 84)

As for barriers to physical activity and exercise, 8.6% cited paresis as limiting, while 35.4% experienced muscle pain post-exercise and 15.9% avoided activity as a result. Lack of motivation or difficulty initiating activity was reported by 22%. Moreover, 10.8% believed physical activity and exercise would worsen their condition, and 16.9% feared it might trigger a disease exacerbation. Figure 1 summarizes selected responses.

### Factors influencing the level of exercise

**Fig. 2:**
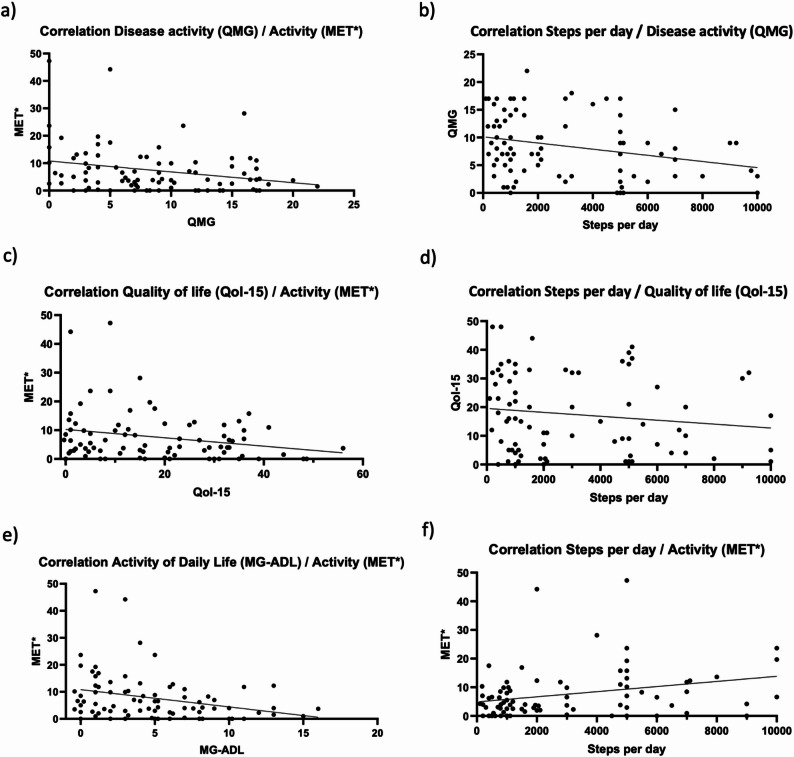
Associations between clinical scores and physical activity. **a** High disease activity (QMG) shows a weak significant inverse correlation with activity (MET*) in individuals with myasthenia gravis, Spearman correlation, *n* = 84, *r* = -0.255, rs = -0.4498 to -0.03603, *p* = 0.019. **b** High disease activity (QMG) shows a weak significant inverse correlation with daily step count in individuals with myasthenia gravis, Spearman correlation, *n* = 80, *r* = -0.265, rs= -0.463 to -0.0415, *p* = 0.018. **c** High quality of life (Qol-15) shows a slight inverse, but not significant correlation with activity (MET*) in individuals with myasthenia gravis, Spearman correlation, *n* = 81, *r* = -0.186, rs = -0.3943 to 0.04005, *p* = 0.096. **d** High quality of life (Qol-15) shows a slight inverse, but not significant correlation with daily step count in individuals with myasthenia gravis, Spearman correlation, *n* = 77, *r* = -0.138, rs = -0.351 to 0.089, *p* = 0.231. **e** Higher impairment in daily activity shows a weak significant inverse correlation with activity (MET*) in individuals with myasthenia gravis, Spearman correlation, *n* = 80, *r* = -0.316, rs = -0.506 to -0.096, *p* = 0.004. **f** High daily activity (MET*) significantly correlates weakly with a higher daily step count in individuals with myasthenia gravis, Spearman correlation, *n* = 80, *r* = 0.313, rs = -0.0936 to 0.503, *p* = 0.005

Physical activity (MET*) and the myasthenia gravis-specific scores show weak inverse correlations (see Fig. 2). Higher disease severity (QMG) was found to weakly correlate with reduced physical activity (*p* = 0.019). Additionally, greater restrictions in everyday activity (MG-ADL) were found to weakly correlate with lower physical activity among individuals with MG (*p* = 0.004). A significant but weak correlation can be demonstrated between daily walking distance and disease severity (*p* = 0.018). Consequently, individuals who exhibited a higher walking distance displayed a mildly lower disease severity. Although a mild correlation was identified between a diminished myasthenia-specific quality of life (as indicated by a higher MG-QoL15 score) and a reduced walking distance (*p* = 0.231) and lower physical activity (MET*) (*p* = 0.096), it did not reach statistical significance (**see** Fig. 2).

### Influence of sex on physical activity and effects of physical activity and exercise

A detailed analysis of the type, frequency and attitude towards physical activity and exercise between the sexes revealed no significant differences overall. The study revealed that males tend to exhibit higher weekly activity (MET* values) (8.3 ± 9.8 vs. 6.5 ± 7.4 for males and females). However, this difference was not statistically significant (*p* = 0.34). Regarding the impact of physical activity and exercise on mood and quality of life, as well as its general physical effects and influence on physical complaints caused by MG, the statements do not show sex-dependant statistically significant differences in the Chi-squared test. It was observed that women experienced heightened levels of muscle pain following physical exertion. However, this discrepancy did not attain statistical significance in the Chi-Square test (*p* = 0.23).

### Influence on motivation on physical activity and exercise

**Fig. 3:**
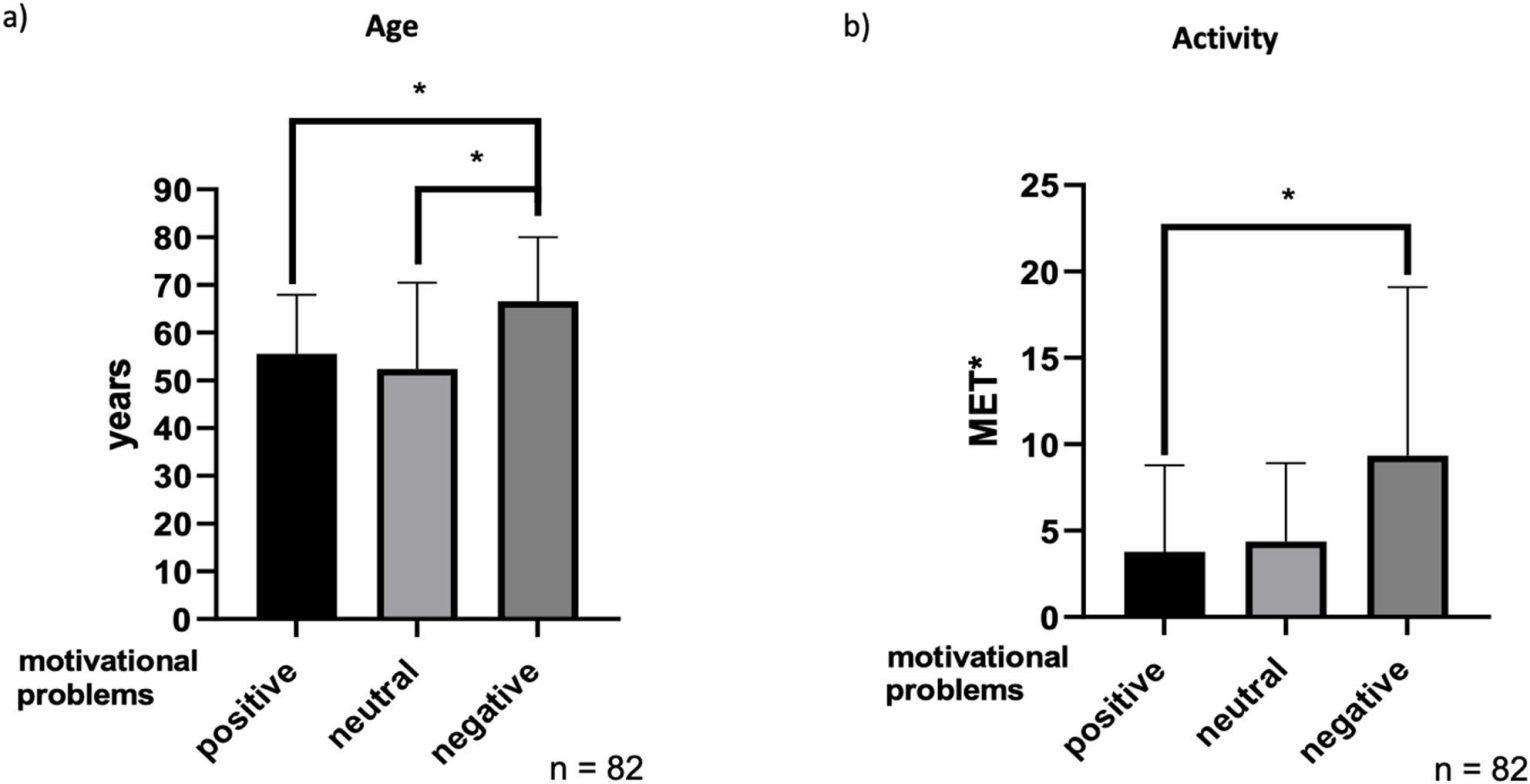
Age and activity in individuals with and without motivational problems regarding physical activity and exercise. **a** Individuals reporting motivational problems are significantly younger compared to individuals without these difficulties. Mean (M) ± SD, One Way Anova, M(positive) = 55.50 ± 12.38, M(neutral) = 52.4 ± 18.09, M(negative) = 66.59 ± 13.39, p(positive - neutral) = 0.837, p(positive - negative) = 0.011, p(neutral - negative) = 0.010. **b** Individuals without motivational problems have a significantly higher MET* compared to individuals reporting motivational problems, Mean ± SD, One Way Anova, M(positive) = 3.78 ± 5.01, M(neutral) = 4.37 ± 4.55, M(negative) = 9.35 ± 9.74, p(positive - neutral) = 0.983, p(positive - negative) = 0.046, p(neutral - negative) = 0.208

As already demonstrated, there is a mild inverse correlation between MG-ADL and QMG and physical activity. Furthermore, most individuals report positive effects of physical activity on their general well-being, mood, and enjoyment of life. However, 22% of individuals also stated that they were unable to bring themselves to engage in physical activity and exercise. A more detailed analysis of this subgroup reveals that individuals with MG who experience difficulties in motivating themselves to perform physical activity and exercise are significantly younger (55.5 years versus 66.6 years, *p* = 0.011). Individuals who are undecided about the statement are significantly younger than individuals who have no motivational problems (52.4 years vs. 66.6 years, *p* = 0.010). However, no dependences on sex or disease duration were observed (see Fig. 3).

Individuals without problems in bringing themselves to perform physical activity and exercise have a significantly higher MET* score than individuals with difficulties (MET* : 9.35 vs. MET* : 3.78, *p* = 0.046). In the subsequent analysis of possible risk factors, it is noticeable that individuals with motivational problems report pain after exercise more frequently, although the Chi-Square test does not show any significant results (*p* = 0.122).

With regard to secondary diagnoses, it is noticeable that the secondary diagnosis of depression (in total *n* = 8) is proportionately higher in individuals with motivational problems compared to those without (22.2% versus 7.4%).

However, no significant differences in QMG, MG-ADL or MG-QoL15 were detected between these subgroups, therefore no differences in severity of illness or everyday life restrictions in individuals with motivational problems regarding physical activity and exercise were observed.

### Influence of pain on physical activity and exercise

A total of 29 out of 84 individuals reported pain after physical activity and exercise. Compared to study participants who did not report pain after physical activity exercise (*n* = 39), a larger proportion of the cohort with pain were female (62.1% vs. 41%). The individuals with pain were slightly younger (58.9 (± 16.5) years vs. 64.6 (± 14.1) years) and had comparable BMIs (28.8 (± 6.2) vs. 28.6 (± 7.5)). In direct comparison with individuals without pain, study participants with pain showed lower activity (measured by MET*), which was not statistically significant. With regard to disease-specific scores (QMG: 10.6 vs. 7.1, *p* = 0.0136; MG-ADL: 6.4 vs. 3.7, *p* = 0.0091 and MG-QoL15: 25.5 vs. 12.7, *p* = 0.007), individuals with pain consistently performed significantly worse.

### Other factors and their impact on activity

In the direct comparison of study participants with the highest physical activity (*n* = 21) with those with the lowest physical activity (also including individuals performing no exercise) (*n* = 21) (upper and lower quartile), no relevant differences were observed with regard to age, sex distribution, BMI, duration of illness, the proportion of myasthenic crises experienced, QMG and MG-Qol15. Individuals demonstrating high levels of physical activity experience significantly reduced impairment in daily functions (MG-ADL) in comparison to those exhibiting the lowest levels of activity (‘high activity’: 3.5 ± 3.5; *n* = 21; ‘low activity’: 6.6 ± 4.5; *n* = 17; *p* = 0.025).

A detailed analysis of the secondary diagnoses revealed no significant differences regarding the presence of lung diseases, joint diseases, spinal stenosis and cardiomyopathies. Interestingly, no individuals reporting a high level of physical activity had a diagnosis of depression (0 of 21 individuals with ‘high activity’ vs. 4 of 21 individuals with ‘low activity’).

Individuals characterized by a high level of physical activity were significantly more likely to report a positive influence of physical activity on their symptoms compared to individuals with a low level of physical activity (57,1% vs. 14.3%, *p* = 0.0028).

## Discussion

Overall, in our study individuals with MG express a predominantly positive attitude toward physical activity and exercise and report perceived benefits for general well-being and mood. However, this study supports previous findings that general physical activity recommendations are often not met in individuals with MG. While a considerable proportion of individuals engage in physical activity more than four times per week, and 37.1% are active for 30–60 min per session, only about one-quarter achieve the World Health Organization’s recommendation of at least 150 min of moderate activity per week. Moreover, only 7.1% of individuals achieved the recommendation for muscle-strengthening activities. However, the extent to which strength components were included within gymnastics and seated gymnastics could not be reliably assessed. These results are in line with other recent studies demonstrating that physical activity levels in individuals with MG fall below both public health guidelines as well as those observed in the general population [18, 19]. Walking was the most commonly reported activity, followed by cycling and seated exercises, types of moderate-intensity activity previously identified as safe and feasible for this individuals affected by MG [13]. Notably, the benefits of walking have already been supported by findings from two independent studies [25, 26].

To date, there is limited comparative research evaluating different exercise modalities in MG. One study compared a home-based training program to spinal stabilization exercises and found the former to be less effective [27]. Another trial comparing progressive resistance training (PRT) with aerobic training showed a slight advantage for PRT [28]. These findings underscore the need for further high-quality comparative studies to determine the most beneficial and practical forms of exercise for individuals with MG.

The safety and beneficial effects of exercise in individuals with MG have been demonstrated in numerous studies assessing various types of exercise across a range of clinical outcome parameters [13–15]. Several studies have shown significant improvements in disease-specific measures such as QMG [29, 30] and MG-ADL scale following exercise [26, 31]. Exercise interventions have also been associated with improvements in functional performance measures. These include increased handgrip strength [29], longer walking distances as assessed by the Six-Minute Walk Test [25, 26], and improved performance in the Chair Rising Test [32]. Furthermore, respiratory muscle training and aerobic exercise have been shown to enhance vital capacity [33], while a 12-week program of combined aerobic and resistance strength training led to an increase in the muscle thickness of the rectus femoris [32]. There is also evidence suggesting that exercise may exert anti-inflammatory effects in MG. A study by Westerberg et al. demonstrated a reduction in pro-inflammatory immuno-microRNAs following a 12-week program of combined aerobic and resistance training [34].

Previous studies have not consistently demonstrated a direct association between severity of functional impairment due to MG and reduced physical activity in daily life [16, 17]. However, other studies with similar designs have shown that more physically active individuals with MG tend to report better MG-ADL scores, improved disease-related quality of life [17, 35], and lower disease severity [18].

In the present study, significant but weak correlations were found between lower disease severity (measured by QMG), fewer limitations in daily living (MG-ADL), and both higher average physical activity levels (measured in MET*) and greater daily step counts. While higher step counts and physical activity levels showed a trend toward better MG-specific quality of life, this association did not reach statistical significance, despite individual responses clearly affirming perceived improvements in mood and quality of life through physical activity.

Birnbaum et al. found no significant improvements in quality of life in either a free-living observational study or a randomized controlled trial involving home-based exercise interventions [31]. In contrast, walking was associated with significant improvements in QOL-15 scores in both observational [26] and interventional studies [25].

When comparing the most and least physically active quartiles of individuals in the current study, no significant differences were observed in disease severity or quality of life. However, limitations in daily living remained significantly different between these groups (*p* = 0.025), suggesting that factors beyond MG severity may influence physical activity behaviour.

This study highlights a notable barrier to physical activity and exercise in the form of motivational difficulties, reported by 22% of individuals with MG. The subgroup with motivational problems was significantly less physically active, regarding the MET* score in comparison to individuals without motivational problem (see Fig. 3b). Aside from a younger mean age and the comparatively higher proportion of individuals suffering from depression, these could not be distinguished by sex or by scores on MG-specific clinical measures.

Anxiety and depression are important comorbidities to consider in individuals with MG [36]. Studies have shown that self-reported MG severity and Quality of life (QoL-15-Score) strongly correlate with symptoms of depression and anxiety disorder in individuals with MG [37]. In this study, outcome parameters for subjective burden of disease as well as quality of life correlated with reduced physical activity and walking distance.

This difficulty in initiating activity may reflect another common symptom of MG - fatigue - which affects approximately 59% of individuals. Fatigue is more frequently reported in women, in individuals with higher disease activity, and in those with comorbid conditions such as depression, and it is associated with substantial psychological and physical burden [38].

Importantly, studies have shown that individuals with higher levels of physical activity tend to report a lower prevalence of fatigue symptoms [18], and that exercise itself can contribute to a reduction in fatigue severity [39], making it as a further cornerstone of therapy in MG.

Despite the availability of innovative immunomodulatory treatment strategies for MG many individuals remain dependent on corticosteroids [40]. Especially during the time of data acquisition (2022–2024), many novel therapeutics were not marketed yet, therefore more conventional treatment strategies, including corticosteroids at the lowest tolerable dose, were commonly applied.

Corticosteroids can potentially severely influence an individual’s psychological situation [41], potentially increasing the risk of mood or anxiety disorders [42]. However, in a healthy population, corticosteroid treatment has been reported to enhance physical performance [42], illustrating the complex effects of this medication on physical activity and overall wellbeing. In rheumatic disease, corticosteroid dosage might be decreased through regular physical activity [43] further emphasizing the intricate interplay between disease state, physical activity and corticosteroid intake.

Engagement in light-to-moderate leisure activities, particularly those involving social interaction (e.g., ballroom dancing, horseback riding, table tennis, or group walking), may improve mood, alleviate fatigue, and enhance muscular strength without inducing symptom exacerbation.

In addition to motivational difficulties, several other barriers to engaging in physical activity and exercise were identified. A substantial proportion of individuals (35.4%) reported pain following physical activity, affecting more women than men and may be associated with poorer disease-specific scores. However, comorbidities such as cardiac or orthopaedic conditions did not appear to significantly limit physical activity within the study cohort. Only a small number of individuals cited misinformation, such as the belief that exercise might worsen their condition, as a limiting factor.

A large-scale study from France also examined perceived barriers to physical activity in individuals with MG. Key barriers identified in that study included unfavourable weather conditions, challenges in balancing physical activity with work and family responsibilities, and fear of symptom exacerbation. Additional factors included poor symptom control, insufficient guidance from healthcare professionals, and a general lack of time [35].

This underscores the importance of distributing high-quality educational materials, for example materials provided by patient lead organisations, to alleviate concerns and demonstrate therapeutic benefits to both patients and their caregivers. Future research should prioritize investigating leisure activities, particularly those involving social interaction, to establish evidence-based guidelines for optimizing engagement and outcomes in MG.

## Limitations of the study

In this study, physical activity was assessed through individual self-reports, which introduces potential reporting bias. Questions regarding exercise duration, frequency, walking distance, and mobility challenges may be subject to overestimation. The use of objective measures, such as pedometers or electronic tracking devices, would have improved data validity and reliability.

The use of MET was appropriate for comparing individuals’ physical activity data, but several limitations of MET must be acknowledged. It is well known that MET values are guidelines insufficiently reflecting age, gender or general fitness. This means that the MET value can be both overestimated and underestimated [44].

Retrospectively, detailed psychometric evaluation of depressive and anxiety symptoms would also have been highly informative. Although no specific testing for these was performed, their presence may confound on the relationship between subjective disease severity and physical activity, as depressive symptoms can lead to both overestimation of somatic symptoms and reduced motivation for physical activity [45–48]. Depression and anxiety are prevalent in MG, with depressive symptoms reported in 19–75% and anxiety symptoms in 17–82% of cases [49, 50]. These psychiatric comorbidities are associated with increased disease severity, reduced quality of life, and higher caregiver burden [51, 52], further underscoring their importance for disease management and patient support.1

While the cohort is representative in terms of age, gender, and AChR-ab positivity, the proportion receiving intravenous immunoglobulin (IVIG) therapy (21.4%) is relatively high. This likely reflects recruitment in 2022, when FcRn antagonists and complement inhibitors were newly approved.

Recruitment at a university hospital specializing in MG may have led to a higher proportion of individuals with complex or active disease, explaining the elevated use of IVIG, FcRn antagonists, and complement inhibitors.

In our cohort, the proportion of AChR-ab-positive individuals who had undergone thymectomy was comparatively low, which initially appeared unexpected. Upon further analysis, we identified that 15 individuals were within the first two years following their initial diagnosis, and 14 of these were AChR-ab-positive. For the majority of these recently diagnosed individuals, thymectomy had already been scheduled but was pending at the time of data collection. This finding reflects standard clinical practice and international consensus guidelines, which recommend thymectomy as an elective procedure for AChR-ab-positive, non-thymomatous generalized MG, typically after establishing optimal symptomatic control and ensuring medical stability in the early phase of disease. Consequently, the lower observed thymectomy rate in our dataset is attributable to the timing of surgical intervention rather than a deviation from established recommendations [53].

No control group was included, as the study aimed solely to assess attitudes toward physical activity in individuals with MG without therapeutic intervention. Comparable studies examining physical activity perceptions [17, 35] or behaviour [54] also omitted control groups.

## Conclusion

Individuals with MG report a subjective positive effect of physical activity and exercise on their general physical well-being and perceived quality of life. Factors such as age, sex, and BMI do not appear to influence the extent of physical activity, whereas a higher symptom burden, and particularly motivational struggles, do. Notably, individuals experiencing motivational issues are not characterized by worse QMG, MG-ADL, or MG-QoL scores, but they do exhibit significantly reduced levels of physical activity. This makes it more difficult for clinicians to identify these individuals.

Since most individuals engage in less physical activity than recommended, healthcare providers should actively inquire about potential barriers and concerns regarding physical activity. Given its beneficial impact on physical condition and its role in reducing cardiovascular risk, individuals with MG should be encouraged and supported in becoming more physically active.

## Supplementary Information

Supplementary Material 1.

## Data Availability

Data are available on request from the corresponding author.

## Abbreviations

Ab: Antibody
AChR: Acetylcholine receptor
AZA: Azathioprine
BMI: Body mass index
F: Female
FcRn: Neonatal Fc receptor
IVIG: Intravenous immune globulin
M: Male
MET: Metabolic equivalent of task
MET*: Modified MET per week
MG: Myasthenia gravis
MG-ADL: Myasthenia gravis-specific Activities of Daily Living scale
MuSK: Muscle-specific kinase
MG-QoL15: Myasthenia gravis-specific 15-item Quality of Life scale
MMF: Mycophenolate mofetil
PRT: Progressive resistance training
QMG: Quantitative Myasthenia Gravis Score
SD: Standard deviation
UMG: University Medical Center Göttingen
WHO: World Health Organisation
