# Supplementary material for: Physical activity and its effects in myasthenia gravis: a patient-reported study on habits and impact
Source: BMC Neurol. 2025 Dec 17;26:38. doi: 10.1186/s12883-025-04590-x (PMC12822234; doi:10.1186/s12883-025-04590-x)
Supplement: Supplementary file 1 — Supplementary Material 1. [file 12883_2025_4590_MOESM1_ESM.pdf]

Questionnaire: ‘Physical activity in patients with neuromuscular diseases’
(This text has been translated from German to English.)

ID: …………..

Please enter the values on the dotted lines and tick the appropriate box.

General information:

Age: ………………………. years Height:………………cm Weight:…………kg

Gender:
o Male
o Female
o Other

Smoking habits:
o Non-smoker
o Smoker

Walking and climbing stairs:
1. What is your maximum walking distance without interruption? (Please estimate your average maximum walking distance that you can cover in one go)

Maximum walking distance: ………………………………… meter

2. How far do you walk each day? (Please estimate your daily walking distance or, if possible, indicate it using a pedometer (smartwatch, smartphone, etc.))

Daily walking distance: ………………………………….. meter

3. **Do you use a pedometer or similar mobility tracker**?
o No
o Yes

4. If so, what exactly do you use?
……………………………………………………………………………………………………
……………………………………………………………………………………………………
……………………………………………………………………………………………………

5. Do you use a walking aid for walking at home?
- o No
- o Single-sided walking aid (walking stick/crutch)
- o Rollator
- o Wheelchair

6. Do you use a walking aid when walking in public areas?
- o No
- o Single-sided walking aid (walking stick/crutch)
- o Rollator
- o Wheelchair

7. How many floors can you climb?
- o None
- o 1 floor
- o 1-2 floors
- o 2-3 floors
- o >3 floors

8. Do you need aids when climbing stairs?
- o No
- o Handrail
- o Handrail and support
- o Stair lift
- o Cannot climb stairs

9. Do you do physiotherapy?
- o No
- o Yes

10. If so, how often do you do physiotherapy per week?
- o Once
- o 1-2 times
- o Twice
- o 2-3 times
- o Three times
- o >3 times

11. How often do you perform inpatient rehabilitation?
- o I have not performed any yet
- o Approximately every 5 years
- o Approximately every 3-4 years
- o Every 2 years
- o Every year

**Frequency of physical activity (Please tick the appropriate box)**					
How many days a week do you exercise?	No day	1 day	2-3 days	3-4 days	> 4 days
How long does a exercise session last?	<10 minutes	<20 minutes	20-30 minutes	30-60 minutes	>1 hour
**What types of exercise activity do you do? (Free text)**					
**To what extent do the following statements apply to you in the last 3 months? (Please tick the appropriate box)**					
	**Strongly disagree**	**Mostly disagree**	**Undecided**	**Mostly agree**	**Strongly agree**
Physical activity and exercise are good for me					
Physical activity and exercise improve my symptoms					
Physical activity and exercise improve my mood/ enjoyment of life					
I can no longer participate in exercise due to paresis					
I have muscle pain after physical					

activity/ exercise session					
	**Strongly disagree**	**Mostly disagree**	**Undecided**	**Mostly agree**	**Strongly agree**
I avoid physical activity/ exercise because of pain					
I am afraid that physical activity/ exercise will worsen my symptoms					
I believe that physical activity/exercise will worsen my symptoms					
Before I became ill, I was very physically active					
I would like to do physical activity/ exercise, but I can`t motivate myself					
Physiotherapy is good for me					
Inpatient rehabilitation is good for me					
